# Relatives’ experiences of care encounters in the general ward after ICU discharge: a qualitative study

**DOI:** 10.1186/s12912-023-01562-9

**Published:** 2023-10-21

**Authors:** Theresa Gyllander, Ulla Näppä, Marie Häggström

**Affiliations:** 1 Department of Health Sciences, Mid Sweden University, Östersund, Sweden; 2 Department of Health Sciences, Mid Sweden University, Sundsvall, Sweden

**Keywords:** Care encounter, Family care, Life-changing events, Nurse–family relations, Nursing care, Quality of care, Relatives, Transitional care

## Abstract

**Background:**

Care encounters at general wards have many reasons, and the complexity differs. Some arriving at the ward are relatives of discharged intensive care unit patients’, who are usually more fragile than others due to what had happened to them. Research indicates that care encounters leave relatives dissatisfied. There is a lack of studies describing how relatives of adult patients experience the transfer from the intensive care unit.

**Aim:**

The purpose of this study was to describe relatives’ experiences of care encounters with nurses during their loved ones’ stay in the general ward after being discharged from the intensive care unit.

**Methods:**

A qualitative descriptive design with an inductive approach was used. Semi-structured individual interviews were conducted with relatives [*n* = 14) of patients from different hospitals in Sweden. Data were analysed using content analysis.

**Results:**

Relatives expressed feeling a huge responsibility for ensuring the quality of care for their loved ones. It was essential to encounter available, committed, and compassionate nurses in the general ward after being transferred from the intensive care unit. The theme ‘*longing for trust and struggling to be involved in their loved one’s care’* was illustrated in the two subthemes of *‘wanting to be seen as an important piece of the puzzle’* and ‘*being vigilant and worrying about the quality of care’.*

**Conclusion:**

The relatives of patients experience their needs as unfulfilled in care encounters with nurses at the general ward after transfer from ICU. In order to meet the needs of relatives, nurses require well-developed non-technical skills to establish a compassionate interaction founded on trust and respect for the individual. Future research should investigate how relatives’ needs can be met in practice. New nursing innovations are necessary to structure encounters with patients and relatives transitioning from the intensive care unit.

## Introduction

Care encounters in general wards have differing complexities for numerous reasons. Some ward patients are discharged from the intensive care unit (ICU); furthermore, these patients and their relatives may often be more fragile than others [1]. Research shows that ICU survivors and their relatives are affected both physically and mentally for a long time after discharge from the ICU [2]. Care encounters are encounters between nurses and recipients of care in caring and connection [3]. It involves nurses encountering the other person as an equal. The consequences of allowing themselves and each other to be the person they are go beyond the actual encounter [4].

## Background

Suddenly finding oneself in a situation where a loved one is critically ill or injured and in need of intensive care can be a life-changing event [5]. Patients who require treatment in the ICU may have been in an accident, undergone surgery or have a serious disease [6] that has resulted in a severe or life-threatening condition. In the ICU, they are connected to life-sustaining medical equipment and closely supervised by a specialist nurse who is part of a multi-professional team responsible for caring for patients and their relatives [7]. Critical illness affects relatives emotionally and physically [5, 8], and they struggle with these problems long after discharge. Research indicates that relatives feel constant anxiety and stress that their loved one may become critically ill again [8].

To be transferred from the ICU to the general ward can be a stressful experience for both the patient and their relatives. The gap between the units includes nursing work patterns, human resources, structural settings, and environmental facilities [9]. Through this transition in care during illness and recovery, patients see their families as providing invaluable support [8].

The quality of the encounters is important. The Fundamentals of Care (FOC) framework outlines three core dimensions for the delivery of high-quality fundamental care: a trusting therapeutic relationship between care providers, care recipients and relatives; integrating and meeting a person’s physical, psychosocial and relational needs; and a care context that is supportive of relationship development and care integration [10]. According to Halldórsdóttir [3], a care encounter is structured on the following three basic components: a caring nurse, connection and a mutual trust between the nurse and the recipient. This is a bridge that symbolises the perceived openness in communication and connectedness with the distance of respect and compassion. On the other hand, if there is no trust or connectedness between the nurse and the recipient, it can be experienced as having a wall in between. A feeling of the encounter as uncaring.

We know that care encounters and a lack of communication leave patients and relatives dissatisfied [11]. However, there are limited studies of how adult patients experience the care encounter with nurses in general wards after being transferred from the ICU. To our knowledge, this study is among the first to describe the complexity of care encounters between relatives and nurses during patients’ stay in the general ward after transfer from the ICU. A deeper knowledge and understanding of relatives’ experiences will guide future improvement frameworks to support nurses in providing quality care in care encounters with relatives. Therefore, the purpose of this study was to describe relative’s experiences of care encounters with nurses during their loved ones’ stay in the general ward after being discharged from the intensive care unit.

## Methods

### Design

This interview study with a qualitative, descriptive design and an inductive approach gives voice to the participants’ experiences and conveys actual happenings. When preparing the manuscript, the Consolidated Criteria for Reporting Qualitative Research (COREQ checklist) was followed [12].

### Participants and data collection

The heads of two ICU were contacted for information about the study, and permission was obtained to ask relatives of former patients to participate. Twenty-two potential participants were contacted via a letter that included a presentation of the study’s purpose and the study’s procedures, allowing them to make an informed decision about whether to participate. Information about the study was spread by participants which produced four additional participants (cf. 13). A sample of 14 relatives—57% female and 43% male [*n* = 14)—participated in the study; four lived in larger cities and 10 in sparsely populated areas in Sweden, which meant that the participants had experience at five different hospitals, different ICUs, and different wards. To capture relatives’ experiences of encountering nurses in the general ward after transfer from the ICU, the inclusion criteria were as follows: (i) having a loved one who had been cared for at the ICU for at least four days during the last three years, and (ii) being able to speak and understand Swedish. Table 1 shows the participants’ characteristics.

**Table 1:** Characteristics of the participants

Characteristics	*n* = 14
Female/Male	8/6
Age range (Md)	40–74 (58)
Relation to the patient	
Spouse Parent Sibling Child	9 3 1 1

Data were collected through individual semi-structured interviews conducted between January and October 2021. A semi-structured interview guide was developed by the authors and tested through a pilot interview before being used. Participants were encouraged to talk as freely as possible about their experiences of encountering nurses in the general ward. Of the 14 interviews, TG conducted 10 and MH conducted 4. The interviews were conducted over the telephone; they lasted between 20 and 68 min (Md 46), digitally recorded and transcribed verbatim. Table 2 presents the questions asked.

**Table 2:** Interview guide

Main questions	Follow-up questions
How was your and your loved one’s first experience with the general ward?	Tell me more about that. How did it make you feel? What made it feel good? What made it feel bad? What did you need in that situation? Can you think of something that could ease the situation? Tell me what happened. Is there anything else you want to tell me?
How were your encounters with the nurses at the general ward?	
Tell me about your needs when encountering nurses at the general ward	

### Data analysis

The interview text was analysed using qualitative content analysis [14, 15], which was mainly conducted by the first author TG in close cooperation with the other authors UN and MH. The text was read several times to gain an understanding of the content and the overall picture before dividing the text into meaning units for decontextualisation. The meaning units were condensed into a description close to the text (manifest content) and abstracted into an interpretation of the underlying meaning (latent content). Recontextualisation refers to taking the condensed meaning units with similar underlying meanings and sorting them together into subthemes. Finally, a theme was identified. The three authors met for several discussions and reflections during the process and went back and forth in the analysis to reach a consensus (cf. 14, 15). Table 3 shows examples of the analysis process. Quotes in the Findings section are presented as those from ‘Relative 1’, ‘Relative 2’, and so on.

**Table 3:** Examples of the analysis process

Meaning unit	Condensed meaning unit	Interpretation of the underlying meaning	Subtheme	Theme
I still feel that we were the closest. They (the nurses) could have received some information from us; for example, ‘He’s like this’ and ‘This is how it is’. Yes, because they asked us a lot of questions at the ICU.	I feel we were the closest. They could receive information from us. At the ICU, they asked us a lot of questions.	Wishing to be seen as a resource by the nurse.	Wanting to be seen as an important piece of the puzzle.	Longing for trust and struggling to be involved in their loved one’s care.
I felt that they (the nurses) did not know these things that they needed to know.	I felt that they had to know.	Feeling concerned about nurse’s ability to provide good care.	Being vigilant and worrying about the quality of care.	

### Rigour

To strengthen the trustworthiness of this study, the authors met for discussion and reflection during the entire process regarding pre-understanding and interpretation. A purposive sample recruiting participants with the best imaginable knowledge of the research question was deemed suitable (cf. 16). To ensure variation and increase credibility, the participants had experience at different hospitals (cf. 14, 16), which was considered to increase transferability to other care contexts (cf. 16). Participants were offered the opportunity to comment on the findings (cf. 14) and confirm the content.

### Ethical considerations

Written and oral information about the study was provided to the participants, who were informed that participation was voluntary, and that they could withdraw at any time during the process until publication without the need for explanation. The participants were guaranteed confidentiality and informed to contact the interviewer if they had any questions.

## Results

**Fig. 1:**
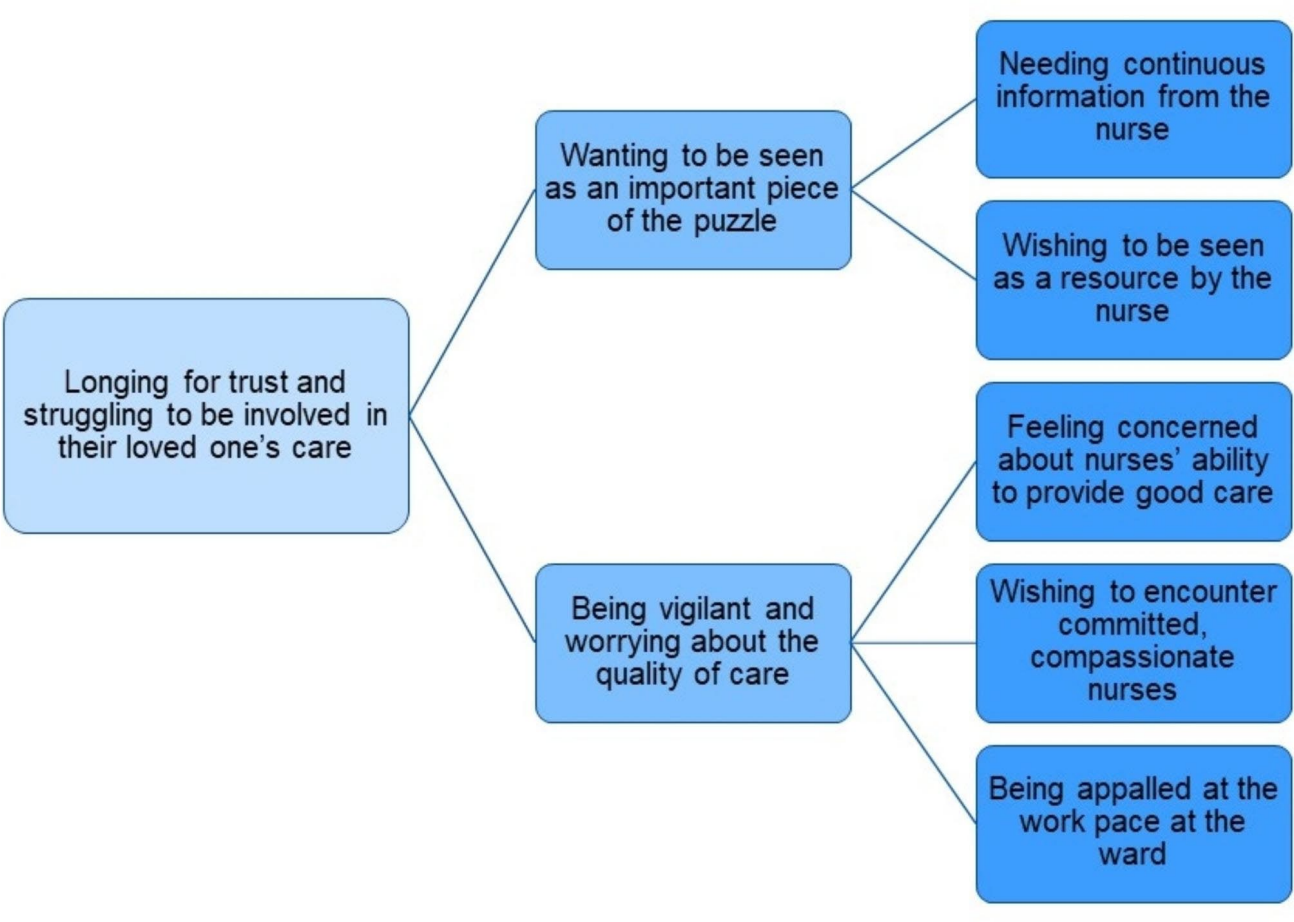
Theme, subthemes and interpretation of the underlying meaning

The overarching theme of *‘longing for trust and struggling to be involved in their loved one’s care’* was illustrated in the following two subthemes: *‘wanting to be seen as an important piece of the puzzle’* and *‘being vigilant and worrying about the quality of care’.* Please refer to Fig. 1.

### Longing for trust and struggling to be involved in their loved one’s care

The theme and two related subthemes together describe relatives’ experiences of care encounters with nurses in the general ward. Relatives felt a huge responsibility to ensure the quality of care for their loved ones during this phase of care. Relatives expressed that after the transfer from ICU, they, as relatives, no longer had a natural place in the care.

They described that encountering committed nurses at the ward was essential to cope with the new situation after the transfer from the ICU. They longed to trust the nurse’s ability and healthcare quality but were often worried about their loved ones. When relatives experienced care encounters in the general ward as deficient, they expressed that they felt anxious, stressed, and vulnerable.

### Wanting to be seen as an important piece of the puzzle

In this subtheme, relatives described how they wanted to be seen as an important piece of the puzzle in the overall care of their loved ones. They were ‘*needing continuous information from the nurse’* and ‘*wishing to be seen as a resource by the nurse´.*

#### Needing continuous information from the nurse

Relatives described that they needed to obtain continuous information from the nurses caring for their loved ones. Information was expressed as an essential part of care encounters, and relatives often made comparisons between how they received continuous information in the ICU. An information flow gave them a feeling of knowing what was going on, whereas the lack of information in the general ward made relatives feel lost.

Relatives felt confused when they experienced a lack of routine and structure in how nurses provided information. They expressed that they constantly needed to ask for information, a struggle that exhausted and annoyed them. It was also challenging to know what information was important to ask for, making them worried about missing out on information.

The quality of the given information was important. Some nurses provided relatives with information contradicting what the other nurses had already told them. This made relatives feel even more confused and anxious when they could not determine who was telling the truth about a specific situation. *‘It is like this. Information about what is happening is the most important thing when you are in the middle of this situation. We need to know why certain things are done’.* – Relative 2. However, relatives said they understood the nurses’ difficulty in informing them when they visited the ward when nurses should be doing other things. Therefore, there was a desire to have predetermined occasions when nurses could provide information about the status and what was happening.

Information about what happened to their loved ones was also performed as care encounters via phone calls. Reliability that nurses would deliver promised information was essential for the relative. They described a wish to get in touch with the nurse in charge by phone at any time of the day to be updated about their loved ones, giving them a feeling of calm and security. However, nurses were sometimes unavailable to provide information, even during phone hours. Relatives described how they were assured by other healthcare personnel that they would be contacted when nurses had the opportunity to do so. This caused stress in relatives as it meant that they would never know how long they had to wait to be phoned back, and it could require several phone calls before being able to get in touch. When relatives were requested to wait and use specific telephone hours, it made them anxious and frustrated, wondering if something bad had occurred. *‘I had to wait until the phone call hour, and it was quite frustrating […] somehow, I was thinking “maybe this isn’t going to be all right….” Not being able to find out, yes, made me anxious.* – Relative 6.

#### Wishing to be seen as a resource by the nurse

Relatives wished to be seen as a resource by nurses and wanted to be welcomed and included as an equal part. They appreciated being given the opportunity to contribute with their own knowledge about their loved one, feeling belonged and as an important part of care. Being involved gave relatives a sense of structure and security and helped them understand what was happening and prepare for their loved one coming home. If they could come and go as they pleased at the ward, it gave them a sense of belonging, calmness, and security. Relatives tried to be a part of the care by sharing ideas and providing suggestions for treatment, demanding that their loved one’s needs should be met. However, they expressed that nurses did not always respond to this in a positive manner. Relatives described occasions when nurses told them not to speculate about treatments when they did not have education in medicine or nursing, which made them feel disconnected.

Relatives also wanted to share their personal knowledge of their loved ones with nurses for better care. They knew personal needs and quickly recognised and saw their loved one’s status and condition. Some relatives were disappointed when they had to give the nurse the same information repeatedly. They meant that the nurse should memorise or include the information in the patient record for other nurses to read. This caused frustration. According to relatives, their information assets were sometimes even neglected by nurses, leading to problems and trouble for their loved ones. *’Why could they not have listened to me earlier? Then, we could have avoided all this trouble’!* – Relative 11. Relatives felt that the care encounter became easier if they had met the same nurse several times. However, relatives described encountering new nurses every time they visited the general ward since the nurses were continuously replaced. *‘There were a lot of different people there I did not know. I recognised them, but I did not remember who they were […] people were replaced all the time’.* – Relative 7.

### Being vigilant and worrying about the quality of care

Relatives described being vigilant, watching over their loved ones and worrying about the quality of care. They were ‘*feeling concerned about nurse’s ability to provide good care’* and ‘*wishing to encounter committed, compassionate nurses.*

#### Feeling concerned about nurse’s ability to provide good care

Relatives who had loved ones being transferred from the ICU were vigilant about the quality of care and felt concerned about the nurses’ ability to provide good care. They described how they appreciated to encounter competent nurses with good communication skills but often met inexperienced nurses who seemed frightened and insecure doing their job. Encountering a competent nurse created a sense of stability and security, which inspired confidence. Furthermore, encountering an experienced nurse who seemed to have control of the situation, performing the tasks correctly, created a feeling that the nurse had the skills required to give the loved one care as needed. When the nurse treated their loved one as a unique person and possessed both medical and nursing knowledge and knew the loved one’s condition, relatives perceived the nurse as competent. Relatives described having to take on the nurse’s role when they thought the nurse lacked the proper skills or time to meet the loved one’s needs. They described how they were helping their loved ones take care of their hygiene, get dressed or get some food. Relatives also expressed fear about their loved one not receiving necessary interventions.

‘I phoned, and the person who answered really did not know. He answered completely wrong, saying that *** was not in pain at all and that there was nothing wrong with the X-ray! I was appalled. It was such a difference compared to earlier at the ICU’. – Relative 12.

They did not dare to leave their loved ones in the ward because they were uncertain whether the care given was what the loved one needed to get better. This felt like a huge, burdensome responsibility and made it difficult for them to trust the care and the nurse. Relatives felt they could not let go of control, which exhausted them.

‘I never forget when this nurse came in. I think she had five or six tablets in a small mug. She just poured it in ***’s mouth and I almost had an outburst […] he had huge difficulties swallowing…. I said, “I know how it has been before and how it should be done, and I thought you had received that information’. – Relative 13.

#### Wishing to encounter committed, compassionate nurses

Committed and compassionate nurses were great important to relatives in the encounters. If the nurse did not exhibit these attributes, the relatives became anxious for their loved one’s condition and were disappointed. A personal touch was appreciated, such as when nurses demonstrated that they knew the loved one as more than just a patient, amongst others. *‘[…] they gave the impression that they knew Dad; that is, they were familiar with him, knew what had happened, and were familiar with the planning’.* – Relative 6. The relatives described committed nurses as those with personal engagement and the ability to listen and show interest, take them seriously, and show compassion, empathy, calmness, and care. They found it highly important that nurses took the initiative to introduce themselves and engage in everyday small talk. Occasionally, some nurses took the time to show their relatives slightly more interest. This was something the nurse did of their own accord and was not part of the routine of the ward.

*‘It feels like it is a bit dependent on the person’s personality. It did not feel like it was something in the routines. I have no memory of that. It is more like it could be someone’s personality that makes you get a different kind of contact’.* – Relative 4.

A lack of personal engagement from nurses made relatives feel sad and unseen. Relatives perceived these nurses as being uninterested and vague. Some nurses gave thoughtless, sarcastic answers to questions, which prompted feelings of unimportance and insecurity in relatives. They felt that these nurses were working only because they had to and not because they were compassionate and wanted to. This contributed to insecurity and feelings of exclusion.

#### Being appalled at the work pace at the ward

When arriving at the general ward, relatives were appalled at the work pace. The transition from the environment in the ICU to that in the ward was perceived as very burdensome by relatives. The environment gave a messy impression, which was almost a shock and made it difficult to encounter nurses. According to the relatives, many people ran around, and the nurses were stressed, always busy and caring for too many patients. It was difficult to gain the nurse’s attention because it was difficult even to find them in sight. *‘It was a different world from the ICU. Here, it felt like they just run around, and do not have time’.* – Relative 1.

## Discussion

This study aimed to describe relatives’ experiences of care encounters with nurses during their loved ones’ stay in the general ward after being discharged from the intensive care unit. The findings demonstrated the overarching theme *‘Longing for trust and struggling to be involved in their loved one’s care’*, further elaborated in the following two subthemes: *‘wanting to be seen as an important piece of the puzzle’* and *‘assessing the quality of care’*. They longed to trust the nurses’ ability and healthcare quality but were often worried about their loved ones.

The overall findings illustrate a stressed healthcare system, with nurses who seems to not have the time to prioritise the relative’s needs. We can also see the vulnerability in the relatives of former ICU patients in terms of them losing the continuous contact to a liable nurse when leaving the ICU. This loss of control seems to make relatives feel even greater responsibility to ensure the quality of care for their loved ones and at the same time experience exclusion from the care after transferring to a general ward can make relatives more vulnerable. The complex care transition between the ICU and a general ward needs to be considered. Successively reducing medical technology and monitoring at the ICU before transfer to the general ward could help both patients and relatives to get used to the lower level of care and make them feel secure. However, earlier research [17] show that ICU-nurses do often not reduce the technology before patient transfer to the general ward because of the culture and the lack of guidance at ICU.

Relatives in this study wanted to encounter compassionate nurses who they believed showed personal engagement, the ability to listen and show interest and empathy. Our study indicates that if relatives are received at the ward by a nurse giving them information and support, as well as involve them in the care, it can provide relatives with feelings of safety when they are torn between hope and despair. When relatives experienced care encounters in the general ward as deficient, they expressed that they felt anxious, stressed, and vulnerable. Even if they were exhausted, it was important for the relatives’ well-being to be able to help and support their loved ones. This seems consistent with the findings of Wong, Redley [6], who showed the importance of relatives in the ICU being there for their loved ones.

The overall findings in our study show relatives’ experiences with caring and uncaring encounters. Nurses need to see beyond being problem solvers or doctoral assistants. They have to bridge the relationship between relatives and caring, according to Halldórsdóttir [3]. If the care encounter is perceived as caring, it can lead to a feeling of creating a bridge of connectedness and openness in communication between nurses and relatives. The findings of our study indicate that relatives perceive nurses in general wards as too busy and disinterested to spend time and energy meeting relatives’ and their loved ones’ needs. At worst, it can create a feeling of a wall between them and the nurse, as Halldórsdóttir describes.

In this study, relatives considered soft values such as good non-technical skills as characteristic of good care encounters. This seems consistent with the findings of Hanssen, Smith Jacobsen [18], who showed that if the nurse respects the other person and defends their dignity with a caring attitude, listens to and keeps the person safe, understands their needs and makes decisions based on them. It is a better chance relatives’ experiencing well-being in a vulnerable situation.

Relatives in this study tried to manage everyday life despite the transition they went through when a loved one suffered a serious event. The overall findings show that it was essential for relatives to have a caring relationship with the nurse in charge of their loved one to be empowered to handle the challenge. Our findings also indicate that relatives may lose trust in nurses when they arrive at the ward after discharge from the ICU. Robinson [19] claims that the most crucial factor for a good care relationship is trust. To be able to make the right care decisions, the nurse must know the patient and their relatives well, and have trusting relationships with them [20]. Therefore, nurses need to be able to create trust with the intention of developing relationships as well as possible. Managing to create good care relationships is partly about nurses’ ability to work with their self-reflection and approach. Adam and Taylor [21] described how nursing students was empowered to develop the quality of relationships by becoming more compassionated. The students received education by a teaching/learning innovation which helped them to develop their emotional strength to deal compassionately with highly emotive situation and recover their strength after emotionally difficult experiences. The education took place in parallel with other teaching. Further training like that ought to be something workplaces should offer nurses.

The findings indicate that if relatives do not receive continuous information, it is hard for them to understand the context of the ward and the care for their loved ones. Leaving the ICU environment, with easy access to nurses, and arriving at the ward where it is difficult even to find a nurse can cause loneliness. This makes relatives more vulnerable, and their need for conversation and information is huge. Nurses must prioritise spending time talking to and informing relatives, which are among their most important duties; healthcare managers should support and encourage this. One way to facilitate this could be to create pathways for receiving and encountering relatives upon arrival at the ward. Compared with other complex care transitions from paediatric intensive care units to paediatric wards, there are transition programmes that decrease and alleviate relatives’ stress and anxiety as well as increase their well-being [22].

Relatives in this study found it essential that nurses took time to introduce themselves, gave information and talked to them in an empathic way, answering questions without any signs of stress. That way, relatives found it easier to feel well-being and contribute to care. This is consistent with earlier research aiming to identify nurse–family relationships [23]. We opine that heads of wards should support nurses in using their worktime to care for and talk to patients and relatives.

The findings of this study indicate the importance of considering the core elements of nursing work when caring for patients and their relatives, to understand their needs, manage fundamental care needs and establish a trusting relationship. This is consistent with the results of Kitson [10], who developed the FOC framework to help nurses provide quality care. Our findings show that it is of great importance that clinical nurses have the resources, right education and intention when encountering relatives to help them maintain well-being despite their vulnerable situation.

### Limitations

The intention of this study was to ensure the participants’ perspectives were accurately represented (cf. 16) and it is important to note that different participants may have yielded different findings and conclusions. The inclusion criteria were having a loved one who had been critically ill and cared for in the ICU; therefore, the diagnosis was irrelevant. Among the relatives who chose to participate in the study, the transfer from ICU to general ward took place for the vast majority within the last year, but for some participants it was up to three years ago. However, it is worth noting that the reliability of memories over time may pose a potential limitation.

Collecting data through semi-structured interviews was found to be an appropriate and credible way to encourage participants to express themselves openly (cf. 14). To enhance dependability, all authors were part of creating the interview guide (cf. 16), consisting of main questions and follow-up questions to capture the nuances of experiences (cf. 14). To facilitate participation in the study, interviews were offered face-to-face or via digital tools. All participants chose telephone. Conducting interviews via digital tools facilitated participation for geographically distant participants. In addition to being far away, previous research [24] has suggested that research participants may find it easier to talk about sensitive issues when they do not have the interviewer face-to-face.

In line with the recommendations of Graneheim, Lindgren, and Lundman [16], we acknowledge that various methods for ensuring trustworthiness are essential throughout the research process. During the planning phase, one critical aspect is the recruitment of participants. Ensuring credibility demands that participants are chosen who are likely to possess relevant experiences related to the studied phenomenon and can articulate them effectively.

An equally vital consideration revolves around the number of participants. As content analysis places a strong emphasis on capturing variations and multiplicity within the data, it is imperative to ensure that there is an adequate amount of data to represent these significant variations. However, it is important to note that specifying an exact number of participants or interviews is not feasible, as the optimal quantity of data depends on the study’s objectives, data quality, and it remains uncertain whether data richness necessarily increases with a higher number of participants or text pages. This point has been emphasized by Sandelowski [25].

In our study, we applied these principles by conducting 12 interviews, and upon observing that the data appeared sufficient to address the research question and encapsulate relevant variations, we conducted two additional interviews. This approach aligns with the aim of achieving both credibility and saturation of data.

All authors were registered nurses and had experience encountering relatives in general wards and ICUs. This pre-understanding was a strength as knowledge influenced the way of meeting these vulnerable participants in the interviews. However, even if the analysis is described as accurately as possible, it is important to note that the qualitative nature of the findings means that replicating the study exactly may not yield identical results, as interpretations play a significant role.

## Conclusion

Relatives of patients transferred from the ICU to general wards experience a strong desire for trust and encounter challenges when attempting to be actively involved in their loved one’s care. Their experiences of unfulfilled needs in care encounters with nurses at general wards unequivocally highlights the need for improvements that enhance the quality of care encounters and promote greater involvement of relatives in patient care. Highly skilled nurses, proficient in non-technical aspects of care, are essential for patients and their relatives as they navigate the recovery process following an ICU stay.

### Clinical implications

Future research should focus on practical strategies to meet the needs of patients’ relatives and explore the systematic implementation of equitable involvement of relatives in patient care. This includes developing clinical pathways for encountering and addressing the needs of patients who have been cared for in the ICU and their relatives. Furthermore, new digital tools that enable nurses to continually update relatives on the condition of their loved ones should be implemented. This underscores the necessity of gaining a deeper understanding of the post-discharge care of ICU patients.

## Data Availability

The full interviews´ data are not publicly available due to privacy or ethical restrictions.
